# Flaxseed Lignans and Polyphenols Enhanced Activity in Streptozotocin-Induced Diabetic Rats

**DOI:** 10.3390/biology10010043

**Published:** 2021-01-11

**Authors:** Dan Draganescu, Calin Andritoiu, Doina Hritcu, Gianina Dodi, Marcel Ionel Popa

**Affiliations:** 1 Faculty of Chemical Engineering and Environmental Protection, Gheorghe Asachi Technical University of Iasi, 700050 Iasi, Romania; draganescu_d@yahoo.com (D.D.); dr_calin_andritoiu@yahoo.com (C.A.); doina.hritcu@gmail.com (D.H.); impopa@yahoo.fr (M.I.P.); 2 Institute of Research and Development in Technical and Natural Sciences, Aurel Vlaicu University, 310025 Arad, Romania; 3 Faculty of Pharmacy, Vasile Goldis Western University of Arad, 310025 Arad, Romania; 4 Advanced Research and Development Center for Experimental Medicine, Grigore T. Popa University of Medicine and Pharmacy of Iasi, 700454 Iasi, Romania

**Keywords:** lignans, polyphenols, flaxseed extract, streptozotocin, antidiabetic effects

## Abstract

**Simple Summary:**

Diabetes mellitus is a metabolic condition that affects millions of people globally. The present study highlights the enhanced activity of flaxseed lignans and polyphenols isolated from *Linum usitatissimum* in streptozotocin (STZ)-induced diabetic rats. Treatment with flaxseed extract showed enhanced glycosylated hemoglobin and blood glucose levels and reduced plasma cholesterol, low-density lipoprotein (LDL) cholesterol and triglycerides, urea and uric acid intensities, and plasma creatinine in the treated diabetic experimental animals, indicating beneficial effects—results sustained by histopathological observations of liver, pancreas, kidney, and spleen. Translation of this nutraceutical-based approach still requires further elucidation of its mechanism of action toward clinical applications.

**Abstract:**

Flaxseeds play an important role in human health due to their chemical composition and recognized beneficial outcomes. This study investigated the antidiabetic effects of present lignans and polyphenols found in the flaxseed extract on streptozotocin (STZ)-induced diabetic rats. The flaxseed administration produced favorable changes in body weight, food and water intake, and glycosylated hemoglobin and blood glucose quantities in the treated diabetic rats. Additionally, significant positive results were observed in the biochemical parameters, namely reduced plasma cholesterol, LDL cholesterol and triglycerides, plasma creatinine, and urea and uric acid levels, highlighting the seeds’ use in traditional medicine. The results were sustained by histopathological observations that showed better tissue preservation following the flaxseed diet. Overall, the consumption of flaxseeds produced moderate reduction in glucose levels and hyperlipidemia, together with improvement in the impaired organs’ function in diabetic rats. The daily administration of polyphenols and lignans compounds could impact therapeutic potential in diabetes management.

## 1. Introduction

Flaxseed (*Linum usitatissimum*), one of the oldest cultivated crops, remains valued for fiber, oil, and food production, and also for potential health benefits [1]. The nutritional composition of flaxseed comprises about 39% oil, 18% protein, and 30% total dietary fiber [2]. The oil is predominantly in the form of triacylglycerides and includes 51–55% α-linolenic acid (ALA) and 15–18% linoleic, 20% oleic, 6% palmitic, and 4% stearic acids [3]. Flaxseed protein contains higher amounts of arginine, glutamic, and aspartic acids [4]. Additionally, it is an abundant source of phenolic products, such as isolariciresinol (ISO), hidroximataresinol (HYDMA), lariciresinol (LARI), matairesinol (MATA), pinoresinol (PINO), secoisolariciresinol (SECO), and secoisolariciresinol diglucoside (SDG) lignans; hydroxycinnamic acid derivatives; acid-4-O-glucoside; ferulic acid-4-O-glucoside; flavonoid herbacetin diglucoside; and *p*-coumaric [5,6,7]. Flaxseed biologically active compounds show a varied range of health-promoting outcomes: antioxidant, antiviral, anti-inflammatory, antidiabetic, and anti-obesity; reduced risk for different cancers (breast, colon, prostate and skin) and cardiovascular disorders; fertility and thyroid function improvement; and menopausal signs and postprandial blood glucose reduction [8,9,10]. Although the beneficial effects of the whole flaxseed or its fractions are well-recognized, it is important to take into account the entire selection of bioactive ingredients in order to associate specific compounds with their respective biological activity.

Diabetes mellitus (DM) is considered a serious and emergent global health problem, with approximately 422 million diabetic people worldwide, which is estimated to be 1 in 11 of the world’s adult population and is expected to increase dramatically up to 592 million by 2035 (International Diabetes Federation). DM is a chronic disease related to metabolic dysfunction initiated by insulin-compromised secretion from pancreatic β-cells, either by insulin synthesis reduction (type I diabetes) or by insulin insensitivity followed later by β-cells’ death in the Langerhans islets (type II diabetes) [11].

In type I DM, insulin is no longer produced by the body since the β-cells of Langerhans islets are destroyed; therefore, high blood glucose levels are encountered along with specific symptoms such as polyuria, polydipsia and polyphagia, blurred vision, fatigue, and dramatic weight loss in a short period of time [11]. Typically, type II DM appears with classical symptoms of sustained hyperglycemia, polyuria, polydipsia and polyphagia, and significant body weight loss caused by structural proteins’ degradation/loss [12], followed by long-term impairment, dysfunction, and breakdown of several organs, particularly eyes, nerves, kidneys, heart, and blood vessels. An efficient approach for DM prophylaxis and treatment is represented by hyperglycemia or hyperinsulinemia correction.

In animal models, DM induction by different methods is currently employed to understand the clinical aspects of its pathogenesis and eventually to find new therapies [13]. STZ (2-deoxy-2-(3-(methyl-3-nitrosoureido) -D-glucopyranose), a DNA alkylating reagent, is often used to mimic diabetes symptoms for type I [14], depicted by pancreatic β-cells apoptosis, resulting in hyperglycemia, diminution of insulin gene expression, and reduced synthesis of insulin [15]. According to the literature studies, type I DM is induced by a single STZ high dose in mice in ranges from 100 to 200 mg/kg and from 35 to 65 mg/kg in rats or by multiple low doses from 20 to 40 mg/kg/day over 5 days in both species [16]. Type II DM animal models often include obese animals, high fat feeding, and β-cell inadequacy [16].

The literature data suggest that flaxseed consumption may protect against this metabolic syndrome by decreasing glucose and lipid concentrations, by postponing postprandial glucose absorption, and by reducing inflammation and oxidative stress [17,18]. Recent studies prove that SDG, due to its known antioxidant activity and its capacity to defeat the gene expression in primary hepatocyte cell cultures, is able to inhibit the DM development in Zucker rat animal models [19]. Hano et al. [20] were the first to propose a mechanism of its action involving the inhibition of the pancreatic α-amylase caused by flaxseed lignans. Moree et al. [21] evaluated the antihyperglycemic endeavor of synthetic SDG in high single dose STZ-induced diabetic rats. The results revealed that SDG significantly reduces hyperglycemia while controlling the blood glucose and serum lipid intensities, regulating the metabolic equilibrium, and maintaining tissue function, leading to improved sensitivity and response of target cells to insulin. Dusane and Joshi [22] highlighted also the antidiabetogenic activity of *Linum usitassimum* fraction (LU6) in single dose STZ-induced diabetic Swiss mice. Daily administration of LU6 indicated beneficial effects in reducing postprandial hyperglycemia, endogenous insulin secretion, and islet regeneration, which can decrease the risk of developing type II diabetes in patients with impaired glucose tolerance. Recently, Kaur et al. [23] assessed the potential of petroleum ether and hydro-alcoholic extract of *Linum usitatissimum* L. in STZ–nicotinamide-induced diabetic nephropathy. The isolated flavonoids, saponins, and terpenoids produced substantial diminution in the glycemic index, level of antioxidant enzymes, renal function, and lipid profile through glycation end products and oxidative stress inhibition [23]. In this context, the present work aimed to investigate the antidiabetic effects of SDG, LARI, MATA, PINO, and SECO lignans and GAE, t-ferrulic, and p-coumaric acids polyphenols administration extracted from commercial flaxseeds upon the clinical and biochemical parameters and histopathological profile in STZ-induced type I diabetic rats.

## 2. Materials and Methods

Flaxseeds (Romanian variety “Cosmin”) were purchased from a local grocery store. STZ (≥75% α-anomer basis, ≥98%), sodium hydroxide, n-hexane, ethanol, citric acid, and hydrochloric acid were purchased from Sigma-Aldrich.

### 2.1. Flaxseed Extract

The flaxseeds were first dried at room temperature in uncovered recipients, grounded to a fine powder in an electric mill (0.5 mm average particles size), and freeze-dried. The powder was placed in a Soxhlet extraction system for solvent defatting using hexane. Next, the defatted powder (ratio of 5:100 (*w*/*v*)) was stirred for 4 h at 60 °C in ethanol/water 70/30 (*v/v*). The extracted solution was cellulose membrane filtered and concentrated (40 °C at 160 rpm) under reduced pressure (175 mbar). The obtained light- yellow syrup was freeze-dried and kept at 4 °C for further use. For HPLC/TOF-MS analysis, the solution was hydrolyzed for 2 h at 80 °C using HCl, neutralized with NaOH, and filtered using a 0.45 µm cellulose filter [24].

### 2.2. Experimental Animals

The Animal Ethical Committee of Grigore T. Popa University of Medicine and Pharmacy of Iasi approved the experimental animal procedures in accordance with the European Community guidelines regarding ethics. Thirty female Wistar rats (8–12 weeks) weighing 240 ± 15 g were supplied by the Cantacuzino Institute. The animals were individually housed (one rat per cage) under standard conditions of 22 ± 3 °C, 60 ± 5% humidity (12-h light/dark cycles) and received food (standard pellet diet) and water ad libitum.

### 2.3. Induction of Experimental DM

The animals were fasted and acclimatized 24 h prior to the STZ injection. Diabetes was induced by a single intraperitoneal injection of STZ solution (12 mg/240 g body mass) in 0.01 M citrate buffer (pH 4.5) according to a slightly modified method [25], standard for type I diabetes development. All animals received 5% glucose solution orally in order to prevent drug-induced hypoglycemia in the early phase. Equivalent amounts of citrate buffer were injected intraperitoneally to the normal rats control group. Blood glucose level was measured through tail tipping using a glucometer (OneTouch SelectTM, LifeScan, Inc., Milpitas, California, United States of America) at 24, 48, 96, and 168 h after STZ administration; levels above 13.8 mmol/L (250 mg/dL) were considered diabetic and included in the study.

### 2.4. Experimental Design

The experimental animals were divided into three groups of 10 rats each: group N normal control (healthy), group DM-C diabetic control, and group DM-T diabetic rats treated with flaxseed extract (daily oral administration of 0.5 g dry material/dispersed in ultrapure water for 60 days). Blood glucose level was measured in fasting animals at a distinctive time frame (1, 2, 3, 7, 14, 21, 28, 35, 42, 49, 56, and 60 days).

### 2.5. Evaluation of Clinical Parameters

During the experiment, the animals were clinically evaluated every 7 days in order to assess the change in body weight in the experimental animals during the treatment period, along with the time variation of water and food consumption.

### 2.6. Biochemical Analysis

After the experimental program, the rats were fasted overnight and euthanized under anesthesia using 100 mg/kg body weight (bw) ketamine administered intraperitoneally. Heart blood was collected in two distinctive tubes: one with EDTA (ethylenediamine tetraacetic acid) anticoagulant for glycosylated hemoglobin determination and another deprived of anticoagulant for serum separation.

The biochemical parameters were carried out in an authorized sanitary veterinary laboratory, Synevovet, Chiajna, Romania. ALT, AST, total cholesterol, LDL cholesterol, triglycerides, and creatinine levels were spectrophotometrically assayed using a Cobas Integra 400 plus analyzer, Roche Diagnostics, Indianapolis-Marion County, Indiana, USA. HbA1c measurements were performed using a standardized immunoturbidimetry method (GSP/DCCT; National Glycohemoglobin Standardization Program/Diabetes Control and Complications Trial reference method) with Cobas Integra 6000 analyzer, Roche Diagnostics, Indianapolis-Marion County, Indiana, USA. HDL cholesterol and serum urea activities were determined by a spectrophotometric assay using the same chemistry analyzer. The uric acid and blood urea nitrogen concentrations were measured using VetTest 8008 serum chemistry analyzer (Idexx Laboratories, Westbrook, Maine, USA).

### 2.7. Histopathological Evaluation

The organs from each group (three animals per group) were removed and stored in 10% formalin solution. Prior to fixation, the pancreas, liver, kidney, and spleen were weighed on a balance to determine the changes in their weight. After 24 h fixation in 10% buffered formalin, the samples were processed: blocked with paraffin, sectioned into 5 µm sections, stained with hematoxylin and eosin (HE), and examined using a clinical microscope (Olympus BX41) with achromatic plane objective (100×, 200× and 400×).

### 2.8. Statistical Analysis

The results were denoted as the mean ± SD values with at least three replications. Statistical analysis was carried out using Microsoft Excel software. The one-way evaluation of variance (ANOVA) and Tukey–Kramer test were used to establish the significant variance between samples. The results were considered statistically significant if *p* < 0.05.

## 3. Results and Discussion

### 3.1. Identified Lignans and Polyphenols Quantification

**Figure 1:**
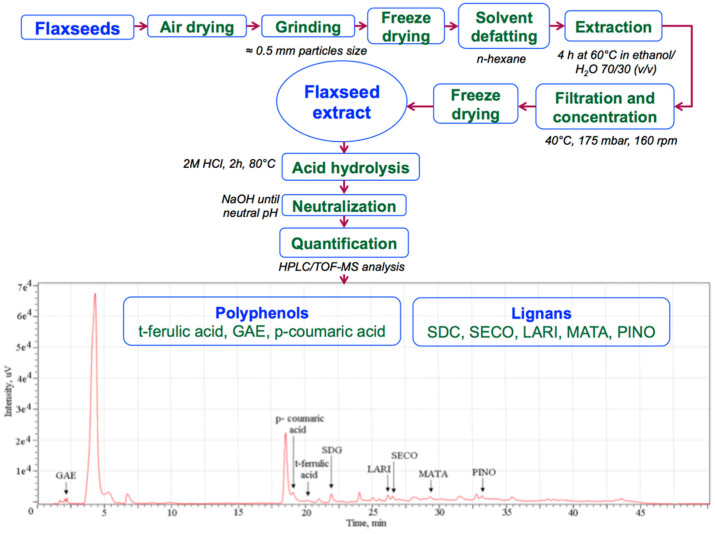
Flaxseed extraction and HPLC/TOF-MS analysis study on HPLC Agilent 1200 Series connected with Agilent 6520 Series Q-TOF-MS electrospray ion source in negative mode.

The identification and quantification of the flaxseed extract compounds by the HPLC/TOF-MS method was performed as published in our previous paper [24] and briefly detailed in Figure 1. SDG, LARI, MATA, PINO, and SECO lignans and GAE, p-coumaric, and t-ferrulic acids polyphenols were identified in the material. The administered dose was calculated based on previous studies that used synthetic SDG in a single dose of 20 mg/kg bw with favorable results [21]. According to Table 1, in 0.5 g of flaxseed extract we separated and identified 0.774 mg lignans and 0.073 mg polyphenols that were administered for 60 days to experimental diabetic rats. STZ-injected rats exhibited standard DM features, such as hyperglycemia, growth retardation, polyuria, renal dysfunction, oxidative stress, and lipid peroxidation.

### 3.2. Changes in Clinical Parameters in Experimental Animals

**Figure 2:**
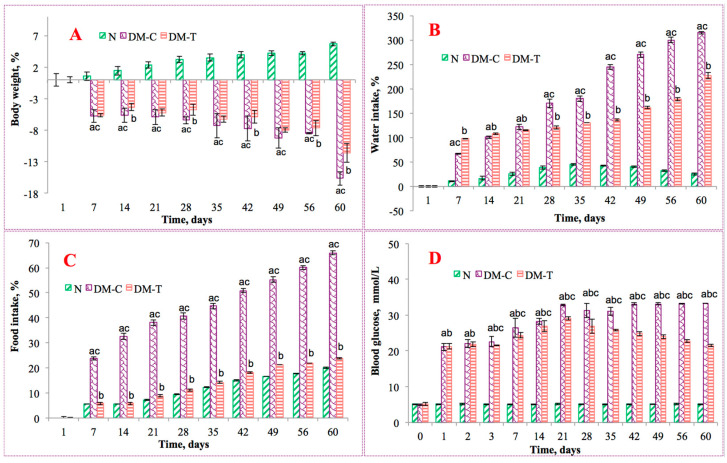
(**A**) Body weight changes; (**B**) water intake; (**C**) food intake, and (**D**) effect of flaxseed extract on blood glucose level in experimental rats. Data are expressed as mean ±SD (*n* = 6/group); *p* < 0.05 using ANOVA followed by Tukey-Kramer test. N, normal control (healthy); DM-C, diabetic control; and DM-T, diabetic treated with flaxseed extract. DM-C vs. N group (a, *p* < 0.05), DM-T vs. N group (b, *p* < 0.05), and DM-T vs. DM-C group (c, *p* < 0.05).

The effects of flaxseed extract on STZ-induced diabetes were observed from the point of view of three clinical parameters, namely body weight changes and the relative intake of water and food in experimental rats. Severe weight loss describes a common feature in DM due to the loss or degeneration of structural proteins. All the diabetic rats (treated and untreated) experienced a loss in body weight at various time intervals, as presented in Figure 2A. The N group showed a slight weight gain of 5.68% when compared with their initial body weight. The DM-C group revealed a reduction in body weight of 15.64% when compared with their initial weight, indicating the effect of metabolic damage caused by STZ. In the case of the DM-T group, only 11.57% decrease in body weight was noticed during the experiment, in agreement with the beneficial properties of flaxseed compounds. The diminished weight loss in the extract-treated diabetic group denotes that the restorative effect of flaxseed may reverse the gluconeogenesis and glycogenolysis developments. These results are in good agreement with the findings from the Florence et al. [26] study that evaluated the antidiabetic activity of *Annona muricata* (Annonaceae) aqueous extract in STZ-induced diabetic rats.

Figure 2B,C displays the impact on water and food intake. In the N group, normal levels of water consumption were measured as 300 ± 21 mL/day. Changes of water intake in diabetic rats varied due to their DM initial symptoms: polydipsia and polyuria. In DM-C, the daily consumption of water was significantly higher (~73%) than the obtained value for the DM-T group, for which the consumption was about 36% higher than N. The additional fluid intake was needed to dilute the high concentration of blood sugar and to compensate for the extra water loss these conditions gave rise to. A similar pattern was observed for food consumption, measured as 99 ± 1 g/day for the N group and 128 ± 10 g/day for the DM-C group, respectively. The treatment with flaxseed extract (DM-T) resulted in a marked decrease in food levels when compared to the DM-C group (23.44%), with a value comparable with N of 98 ± 3.2 g/day.

The obtained results evidenced the positive effects of flaxseed extract upon the monitored clinical parameters, specifically in water consumption volume, which was higher in the untreated group. Although the food consumption level was significantly higher for the DM-C group, no increase in body weight trend was observed during the experiment, which could be correlated with the intake.

### 3.3. Biochemical Analysis

STZ caused diabetes by rapid depletion of β-cells, followed by a marked degeneration of the Langerhans islets and reduced insulin synthesis, thus producing a drop in the rate of glucose to glycogen conversion, ultimately leading to hyperglycemia. Daily administration of flaxseed extract produced favorable changes in biochemical parameters related to glycemic control, hepatic and renal function, and lipid profile.

Figure 2D displays the blood glucose levels of all groups over the 60-day treatment period. The blood glucose levels in the N group varied insignificantly, while for the DM-C group a significant increase in blood glucose compared with N group was observed. Following flaxseed extract oral administration, the blood glucose level was significantly reduced for the DM-T group when compared to DM-C. This reduction was not large enough to bring them to normal level, but it was still significantly higher—suggesting that the extract contains biologically active principles, such as tannins and flavonoids with potent hypoglycemic properties. This is probably related to the amplified insulin synthesis, amplified glucose peripheral utilization, inhibition of endogenous glucose production, or by intestinal glucose absorption inhibition, as reported by Doyen et al. [27]. The extract may also have potentiated pancreatic secretion of insulin by the current residual pancreatic β-cells.

To characterize the metabolic ability of flaxseed extract-treated rats, serum HbA1c levels were measured for all experimental animals and are presented in Table 2. HbA1c reflects average plasma glucose levels over extended periods of time with the recommended cut point concentration for diagnosing diabetes of 53 mmol/mol. The increased HbA1c content in the DM-C group (503.57% higher than N group) indicated inferior control of blood glucose quantities associated with cardiovascular disease, retinopathy, and nephropathy [28]. Daily treatment with flaxseed extract improved the HbA1c levels in DM-T as compared with the DM-C group (a decrease of about 7.36%), indicating that the extract works as a hypoglycemic agent. The HbA1c value increase correlated with the blood glucose level. Therefore, administration of flaxseed extract to diabetic STZ-rats reduced the glycosylation of hemoglobin by virtue of its normo-glycemic activity and thus increased the levels of hemoglobin in diabetic rats.

Elevated activities of serum aminotransferases, frequent in diabetes, are a common sign of liver disorders and are linked with diabetic problems such as retinopathy, neuropathy, and limited joint mobility and are also linked with modified liver enzyme activities, independent of body mass index and metabolic control [29]. The levels of the biomarker enzymes AST and ALT as indicators of tissue toxicity were higher in DM-C over a 60-day period, indicating the hepatocellular damage when compared with nondiabetic rats (N). After flaxseed extract daily administration, the liver tissues of treated diabetic rats denoted the same development toward amplified transaminases activity as the diabetic control (DM-T). The increased gluconeogenesis and ketogenesis could be due to amino acid availability in the diabetic blood since the transaminases are active in the absence of insulin. The insignificant reversal of ALT and AST activity in flaxseed-treated diabetic rats toward near normality represents a confirmation of the tissue and cellular damage under diabetic settings, which may further boost liver metabolism changes in diabetic rats. From this point of view, flaxseed extract did not act as a hepatoprotective agent as expected; therefore, more insights will be observed in the histopathological studies. Our results support those reported by Doss et al. [30] regarding the fact that transaminase activity is increased in diabetic rats’ serum treated with *Solanum trilobatum* (Linn.) aqueous extract.

Hypertriglyceridemia and hypercholesterolemia are the most commonly recognized complications of DM and are described by elevated cholesterol, triglycerides, and phospholipids levels and also by lipoprotein composition changes. Therefore, the ideal treatment of diabetes, in addition to glycemic control, should also have a favorable effect on lipid profiles.

The effects of flaxseed extract on total cholesterol, HDL cholesterol, LDL cholesterol, and triglyceride levels are shown in Table 2. The concentration of lipids, such as serum total cholesterol, LDL, and triglyceride, were significantly higher in DM-C than those in N rats (increase of 132.8% for total cholesterol, 56.98% for LDL, and 134.5% for triglyceride), with the exception of HDL (indexes of lipid peroxidation) that had a comparable value in both groups. A variety of imbalances in metabolic and regulatory mechanisms due to insulin deficiency may be responsible for the observed accumulation of lipids. Daily administration of flaxseed extract (DM-T group) reduced the total cholesterol, HDL, LDL, and triglyceride levels significantly when compared with DM-C rats (decrease of 36% for total cholesterol, 16.81% for HDL, 5.93% for LDL, and 36.81% for triglyceride). The results are attributed to its protective role upon membrane-bound lipoprotein lipase (an enzyme involved in triglyceride hydrolysis that is reduced in diabetes) against oxidative degradation of lipids [31], beneficial in preventing diabetic complications as well as in improving lipid metabolism in diabetics. This promotes antihypertriglyceridemia.

Future perspectives for study of the effects of flaxseed extract upon STZ-diabetic rats could involve important parameters, such as quantitation of triglycerides in the liver, the presence of glycogen using PAS (Periodic acid–Schiff), proteinuria, insulin sensitivity, or urinary excretion of glucose, in order to elucidate the possible mechanism by which flaxseed extract produces favorable results.

The recorded changes in renal function [32] after the treatment are shown in Table 2. STZ (DM-C group) induced increased values of serum creatinine, serum urea, uric acid, and blood urea nitrogen level when compared to the N group, indicating a reduced ability of the kidney to filter them from the blood and excrete them in the urine. The higher levels of blood urea nitrogen and serum creatinine suggests progressive renal damage. On the other hand, administration of flaxseed extract significantly reduced creatinine, serum urea, uric acid, and blood urea nitrogen levels in DM-T when compared to the DM-C group, a process that could be related to gluconeogenic amino acids participating in gluconeogenesis. These improvements attributed to flaxseed extract enhanced the renal function and delayed progression of DM toward the end-stage, namely renal failure. Based on the obtained results, the flaxseed extract may boost the kidneys’ ability to remove from the blood these unwanted products, as indicated by a protective effect on the diabetic rat kidneys.

### 3.4. Observations of Organs

The mean values of liver, pancreas, kidney, and spleen weights were determined in all groups and are shown in Table 2. Liver weight was slightly amplified in DM-C rats when compared with N rats (increase of 9.8%), but after the repeated flaxseed extract administration the weight decreased to a tolerable normal value. The growth (hypertrophy) in liver weight is attributed to increased triglyceride accumulation induced by hypoinsulinemia and the low capacity of excretion of lipoprotein secretion from liver.

Pancreas weight was slightly decreased in DM-C (10%) and slightly increased in the treated diabetic rats (10% for DM-T) when compared with the N group. The shrinkage in the pancreas weight could be attributed to the disruption and disappearance of pancreatic islets and selective destruction of insulin-producing cells [33]. These results are in good correlation with the histological studies.

Kidney weight was significantly increased in the DM-C group when compared with N of about 78.89% and DM-T of about 46.26%. The noted increase in the DM-T kidney weight, despite the fact that the mean of all the animals in the STZ-induced diabetic group diminished, suggests the presence of renal hypertrophy. According to the literature data [34], the renal hypertrophy might be due to a reduction in the renal extracellular matrix components, tubulointerstitial fibrosis, and fibronectin degradation, as observed in the histopathological examination. Spleen weight in all groups was practically unaffected by the STZ or by the flaxseed extract administration.

The weights of examined organs showed that STZ caused cellular necrosis and selective destruction of the pancreatic β-cells in diabetic animals and was linked with the relative weight of the organ’s increase—an effect that was reversed by flaxseed extract administration to appropriate values characteristic of healthy animals. These findings are in good agreement with those reported by Sundaram et al. [35].

**Figure 3:**
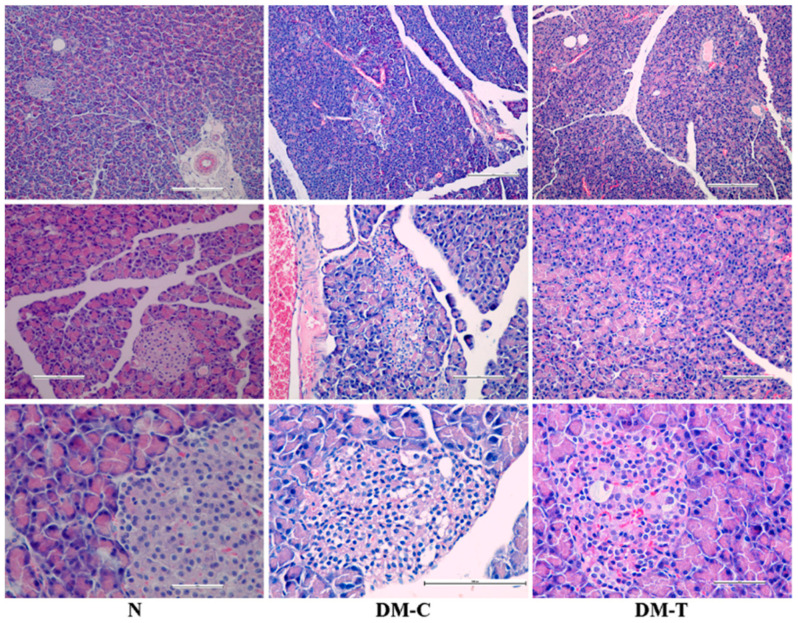
Histopathologic examination of pancreas in N, DM-C, and DM-T rats.

The cytoarchitectural changes of pancreas are presented in Figure 3 and are described below:

- N group: Complete normal islet structure with regularly distributed and abundant pancreatic cells.
- DM-C group: The pancreatic islets morphology showed pronounced endogenous damage, such as cytolysis, cell rupture, and apoptosis; the Langerhans islets number was reduced and accompanied by the cytoplasmic vacuolation of pancreatic β-cells, responsible for secreting insulin and vascular congestion; histopathology examination revealed the presence of an infiltration with lymphocytes and mastocytes located at vascular–conjunctive interlobular septum.
- DM-T group: The treatment with flaxseed extract reverted the abnormal modifications in islet structure located in the STZ lesions, contributing to architectural amelioration of islet structure and a gradual increase in β-cell counts; the number of damaged pancreatic Langerhans islets decreased substantially after the flaxseed extract administration. A small percentage of the pancreatic β-cells still preserved the cytoplasmic vacuolation, accompanied by a reduced infiltration with lymphocytes and mastocytes at the vascular–conjunctive interlobular septum. The obtained architecture implies the possibility that flaxseed extract is capable of protecting pancreatic β-cells or promoting their regeneration.

Further research could be done, especially with reference to the number and diameter of the pancreatic islets, such as immunohistochemistry analysis to examine the expression of α and β cells upon treated diabetic rats.

**Figure 4:**
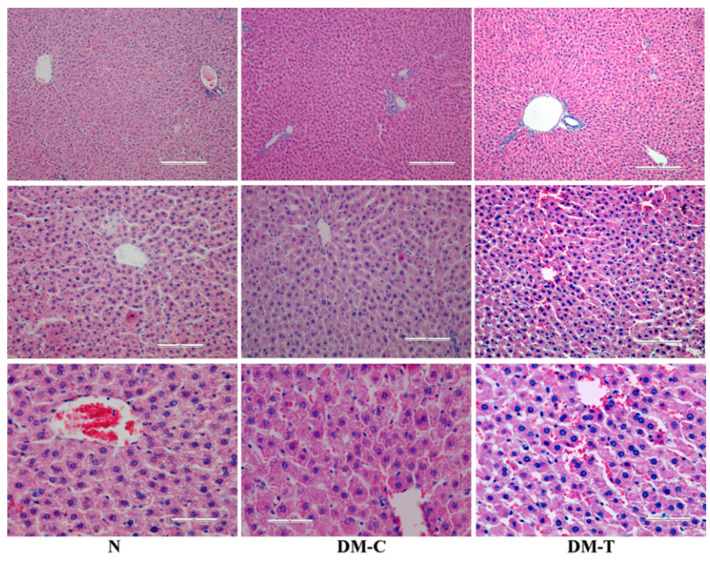
Histopathologic examination of liver in N, DM-C, and DM-T rats.

The histopathological examination of liver is shown in Figure 4:

- N group: Intact architecture and normal hepatocytes with well-preserved cytoplasm, nucleus, nucleolus, and central vein.
- DM-C group: The hepatic cords became distorted and the hepatocytes evidenced a low deposit of glycogen, in association with necrosis of the hepatic cells, degeneration, vacuolation in hepatic cells, and infiltration of the parenchyma with inflammatory cells in comparison to healthy rats.
- DM-T group: Flaxseed extract administration significantly tempered the STZ-induced hepatocellular necrosis and fibrotic changes in rat liver; the mild normalization of hepatocellular architecture with nucleus, cytoplasm, and distinct hepatic layer was also observed; on the other hand, the reduced capacity of liver to produce glycogen was maintained [36].

Similar interpretations were described when using parsley (*Petroselinum crispum*) aqueous extract that reduced hepatocytes degenerative changes in diabetic rats [37].

**Figure 5:**
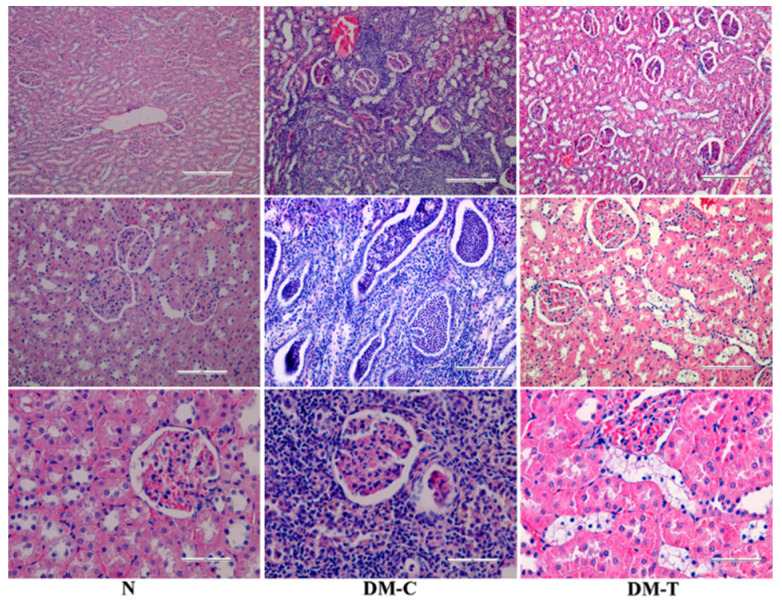
Histopathologic examination of kidney in N, DM-C, and DM-T rats.

The kidney morphology appears in Figure 5:

- N group: Normal structure of renal tubules, renal corpuscles, and collecting ducts without any inflammatory changes.
- DM-C group: Showed infiltration of inflammatory cells (neutrophils, lymphocytes, and plasmocytes) spread as an unlimited band in the cortical region up to renal pelvis. The obliterated lumen from neutrophils accumulation of renal tubules was observed in association with multiple areas of necrosis involving glomeruli, the renal tubules, and the walls of the blood vessels. The renal pelvis exhibited edematous changes with cellular injury and moderate lymphocytes infiltration at subjacent transition epithelium region. Additionally, the necrotic changes displayed hemorrhage in renal cortex, an increased space filtering, compression atrophy of the renal glomeruli, and dilatation of the renal collecting tubules. All of these features indicate a suppurative nephritis and renal hydronephrosis.
- DM-T group: The atypical modifications in renal structure were partially rescued in the STZ lesions, contributing to architectural amelioration of epithelium necrosis and displaying features of epithelium necrosis with a reduced number of renal collecting tubules.

**Figure 6:**
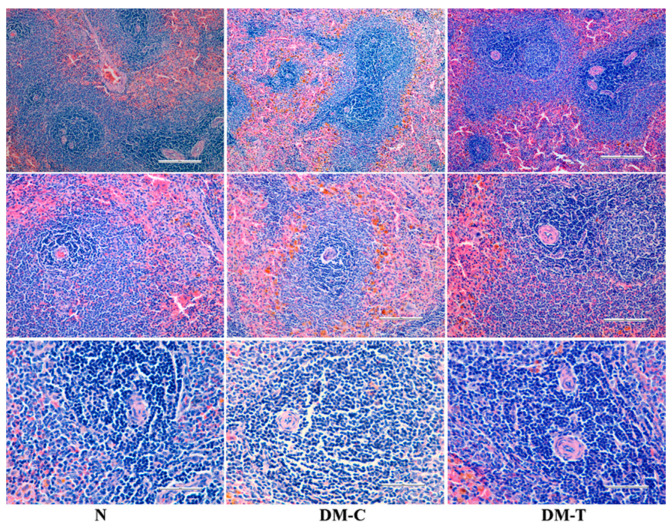
Histopathologic examination of spleen in N, DM-C, and DM-T rats.

Histopathological observations in spleen from Figure 6:

- N group: A highly vascular organ with red pulps represented by blood sinuses and white pulps indicated by oval splenic discrete nodules of lymphoid accumulations adjacent the central arteries, creating periarteriolar lymphoid sheath.
- DM-C group: Displayed normal architecture comparable with the healthy rats.
- DM-T group: No injuries on spleen tissue were observed for diabetic rats treated with flaxseed extract.

In accordance with the observations on the measured organs’ weights, the present-noticed positive reaction of the spleen of treated diabetic rats may be an indication of non-toxicity for rats in acute administration of flaxseed extract.

## 4. Conclusions

Overall, the presented results display that flaxseed extract can serve as a promising additional therapy approach in managing diabetes due the quantified effects: reduced blood glucose levels, body weight loss, and food and water intake and improved lipid and renal profile. Additionally, the histopathological investigations showed that flaxseed extract partially recovers pancreas, liver, and kidney functions, thus reducing the lesions associated with the diabetic state. Therefore, the administration was successful in reducing blood glucose, lipid profiles, and histopathology of pancreatic β-cells, although it did not bring them to normal levels after 60 days. The potential way that the values could be brought closer to normal could be either by dose–response studies and multiple markers measurement (proteinuria, insulin sensitivity, or urinary excretion of glucose) or by multiple STZ low-dose administration for fewer toxic effects and therefore an enhanced effect of flaxseed extract. The present study creates further opportunities to separate the active constituents from flaxseed that are accountable for antidiabetic activity and to clarify their mechanism of action. More studies are required that drive together primary preclinical and clinical results with standardized product formulations development to optimize the dose of polyphenols and lignans within an edible product that could be administered along with classic therapy.

## Figures and Tables

**Table 1:** Quantitative calculation of lignans and phenolic fractions from flaxseed extract.

Compound		Concentration/100 g Dry Material	Daily Administrated Concentration/0.5 g	Total Administrated Concentration/30 g
Lignans	SDG	9.3 mg	0.774 mg	46.44 mg
	SECO	110.8 mg		
	LARI	4.9 mg		
	MATA	6.9 mg		
	PINO	22.9 mg		
Polyphenols	*t*-ferulic acid	4.8 mg	0.073 mg	4.38 mg
	GAE	4.6 mg		
	*p*-coumaric acid	5.2 mg		

**Table 2:** Quantitative biochemical parameter levels in experimental rats and mean weights of pancreas, liver, pancreas, and spleen at sacrificial time in experimental animals. Data are expressed as mean ±SD (*n* = 6/group). Statistical significance of DM-C vs. N group (^a^, *p* < 0.05), DM-T vs. N group (^b^, *p* < 0.05), and DM-C vs. DM-T group (^c^, *p* < 0.05).

Parameter/Group	N	DM-C	DM-T
HbA1c, mmol/mol	18.47 ± 0.1	111.48 ± 0.3 ^ac^	103.27 ± 0.4 ^bc^
AST, UI/L	94.5 ± 4.95	378.5 ± 17.68 ^a^	374 ± 16.97 ^b^
ALT, UI/L	44.5 ± 3.54	176.5 ± 4.95 ^ac^	182.5 ± 3.53 ^bc^
Total cholesterol, mg/dL	62.5 ± 3.53	145.5 ± 4.95 ^ac^	93 ± 2.83 ^c^
HDL cholesterol, mg/dL	59 ± 1.41	59.5 ± 4.95	49.5 ± 3.53
LDL cholesterol, mg/dL	43 ± 4.24	67.5 ± 3.54 ^a^	63.5 ± 2.12 ^b^
Triglyceride, mg/dL	100.5 ± 14.85	235 ± 14.14 ^ac^	148.5 ± 4.95 ^c^
Creatinine, mg/dL	0.175 ± 0.05	0.13 ± 0.07	0.095 ± 0.02 ^bc^
Serum urea, mg/dL	53.5 ± 2.12	75 ± 1.41 ^ac^	58 ± 4.24 ^c^
Uric acid, mg/dL	1.05 ± 0.35	2.05 ± 0.5 ^ac^	0.7 ± 0.57 ^c^
Blood urea nitrogen, mg/dL	25.17 ± 0.24	34.97 ± 0.76 ^ac^	27.02 ± 1.97 ^c^
Weight of pancreas, g	0.6 ± 0.01	0.54 ± 0.03 ^ac^	0.66 ± 0.01 ^c^
Weight of liver, g	10.2 ± 0.01	11.2 ± 0.05 ^ac^	9.92 ± 0.61 ^c^
Weight of kidney, g	0.9 ± 0.005	1.61 ± 0.21 ^ac^	1.1 ± 0.07 ^c^
Weight of spleen, g	0.55 ± 0.005	0.53 ± 0.08	0.57 ± 0.005

## Data Availability

Not applicable.
